# Effects of High Frequency Chest Wall Oscillation (HFCWO) on Clinical Symptoms in Chronic Obstructive Lung Disease

**DOI:** 10.18103/mra.v12i11.5805

**Published:** 2024-11

**Authors:** Meaghan M. Bruner, Clarissa Bazan, Bo Liu, Christina Cheng, Chad Marion, Chet Sievert, Lloyd J Edwards, George M. Solomon

**Affiliations:** 1 University of Alabama at Birmingham, Birmingham, AL USA.; 2 Wake Forest University School of Medicine, Winston-Salem, NC USA.; 3 Clinical Research Associates LLC, Mahtomedi, MN USA.

**Keywords:** COPD, mucous hypersecretion, mucociliary clearance, high frequency chest wall oscillation, HFCWO, The St. George’s Respiratory Questionnaire for COPD Patients

## Abstract

**Background:**

Mucociliary clearance plays a critical role in pulmonary host defense. Abnormal mucociliary clearance contributes to the pathogenesis of pulmonary disorders, including COPD. In bronchiectasis, treatments targeting mucus obstruction in the airways include the use of high frequency chest wall oscillation (HFCWO) therapy. This prospective outcome based study was designed to investigate the changes in symptoms and quality of life (QOL) to measure the effect of adjunctive HFCWO therapy to standard of care therapy for patients with COPD.

**Research Question:**

When HFCWO is indicated and used as intended, will the quality of life for those patients with COPD improve and sustain improvement.

**Study Design and Methods:**

We conducted a prospective, openl-label, observational study in COPD patients without concomitant bronchiectasis. Participants had assessments of QOL at baseline (day 0) and then at 30 and 90 days after initiation of HFCWO therapy. The St. George’s Respiratory Questionnaire for COPD Patients (SGRQ-C) was employed and longitudinally followed at each timepoint. Paired t-tests were used to compare means between each time points adjusted for multiple comparisons. A linear mixed model for the analysis of longitudinal data was then constructed to determine the simultaneous contribution of race, gender, ethnicity, time, and selected interactions in the primary outcome of change in SGRQ-C across 0, 30, and 90 days.

**Results:**

The cohort of patients (n=102) demonstrated a significant reduction in the SGRQ-C at 30 and sustained at 90 days compared to baseline. In addition, two component scores of the SGRQ-C questionnaire (“Symptoms” and Impacts”) were significantly reduced at 30 and 90 days.

**Interpretation:**

This prospective, observational study demonstrates statistically significant and clinically favorable responses to HFCWO as an adjunctive therapy for patients with a primary diagnosis of COPD without concomitant bronchiectasis. Results of this study inform the design of additional additional studies of HFCWO to prove efficacy in COPD patients.

## Introduction

The lung is continuously exposed to particles, toxins, and pathogens dealt with by the localized innate system for clearance, a key aspect of host defense. Mucociliary clearance plays a critical role in a multilayered defense system and abnormalities in mucociliary clearance contribute to the pathogenesis of many common pulmonary disorders. In Chronic Obstructive Pulmonary Disease (COPD) mucociliary clearance is impaired by various mechanisms^1^. One proposed mechanism responsible for excessive mucus production is the overproduction and hypersecretion by goblet cells^2,3^ and mucus hyperviscosity and hyper-concentration^4^. COPD patients also experience difficulty in clearing secretions due to distal airway occlusion, poor ciliary function, and ineffective cough^2^.

Mucus burden is also a major contributing factor in the morbidity of COPD patients. The rate of decline in lung function, which is a strong predictor of morbidity and mortality for COPD, as well as health care utilization, is higher in patients with mucous hypersecretion^5,6^. One study looked at cases where COPD was an underlying or contributory cause of death and found that patients with COPD and chronic mucus hypersecretion are more likely to die from pulmonary infections compared to patients without chronic mucus hypersecretion^7^. A recent landmark paper examined the effects of mucous plugs on all cause mortality in patients with COPD and found that patients with mucous plugs occluding medium-to large sized airways had an increased all cause mortatliy^8^. There are various methods to improve mucociliary clearance, however little is known about the effect of mucus targeted therapies in COPD as many of the approved pharmacologic therapies are primarily anti-inflammatory or bronchodilators. In general, however, recent investigations have found that healthcare providers are aware of methods and agree with a potential benefit in some patients with COPD^9,10^. There are several therapies that target elements of impaired mucociliary clearance including goblet cell hyperplasia. These inbestigational therapies include: bronchial rheoplasty which shows promise to reduce the production of mucus from secretory (goblet cells) in chronic bronchitis and has had early success in clinical trials^11,12^, metered cryospray and targettedlung denervation^13,14^.

In other airway diseases such as CF and non-CF bronchiectasis (NCFB), many treatments target improving mucus obstruction in the airways. Pharmacological interventions include expectorants, mucolytics, and mucokinetics^10,15^. Non-pharmacological modalities include chest physiotherapy, postural drainage, chest wall percussion and vibration, forced expiration, positive expiratory pressure devices, and high frequency chest wall oscillation (HFCWO)^15^. HFCWO therapy is delivered to the patient’s external chest wall via an inflatable vest that imparts oscillating pressurized air pulses that impart compression forces while also generating high-velocity expiratory airflow that are reported to shear and mobilize mucus from the airway walls thereby improving airway mucus clearance^16^. HFCWO therapy is often used in conjunction with mucolytics and antibiotics in bronchiectasis and CF patients to improve mucus burden. Several studies in CF patients found HFCWO therapy as effective as or superior to other airway clearance methods^15,17^. It has been previously shown in a prospective study that in patients with non-cystic fibrosis bronchiectasis (NCFB), the initiation of HFCWO was associated with reductions in patient-reported exacerbation rates, hospitalizations, antibiotic use, and improvements in respiratory symptoms and quality of life^15,18^.

More recently, patients with COPD have gained reimbursement from Medicare to utilize HFCWO. Although this isn’t yet a guideline-based therapy, HFCWO can be used in addition to other guideline-based therapies for COPD. Given the burden of mucus in many patients with COPD, we hypothesized that HFCWO therapy used regularly would improve patients’ quality of life. Thereby, we designed this quality of life based study to investigate the clinical outcomes of using HFCWO as an adjunctive therapy for COPD as measured by using the St. George’s Respiratory Questionnaire for COPD Patients (SGRQ-C) which is a disease specific instrument designed to measure the impact on overall health, daily life, and perceived well-being in patients with COPD^19^. This has been an extensively validated questionnaire and often used to evaluate the advantage of a new treatment for COPD^19,20^.

HYPOTHESIS: Based on this background, we hypothesized that HFCWO, when used according to intended and indications for use will improve the quality of life for those patients with COPD and the symptomatic improvements will be sustained.

## Study Design and Methods

### STUDY DESIGN

This non-randomized, prospective, open-label, observational study (NCT04271969, Registration Date 18/02/2020). Patients with COPD without bronchiectasis were recruited after HFCWO therapy was prescribed by their attending physician for their clinical condition. Patients were selected by their primary physician for enrollment. Patients were enrolled based on perceived mucus hypersecretion and were felt to be at high risk for exacerbation. The COPD diagnosis was made by the participant’s physician. Patients were excluded that had evidence of bronchiectasis on high resolution CT scans done within 12 months of the study, based on prescribing physician assessment. The study was done during the Covid-19 pandemic, plus the decentralized method and nature of the study design limited some of the information obtained regarding patients COPD diagnosis.

To achieve enrollment, a list comprising the continental United States was collected from the study device (SmartVest) manufacturer. The list of patients was reviewed by study trained Respiratory Therapists (RT) from Electromed, Inc and an independenly employed clinical research associateand from an independent clinical research organization firm to minimize bias. The CRA determined if the participants met study inclusion/exclusion criteria and were willing to participate in the study. Other inclusion criteria included a confirmed COPD diagnosis accompanied by a SmartVest prescription in patients at least 18 years old. Exclusion criteria excluded those currently using HFCWO therapy and aforementioned physician assessment of concomitant bronchiectasis. If enrollment criteria were met, the Electromed RT delivered the device, performed traininng and obtained an informed consent and the patient was enrolled as a participant for phone calls by the independent CRA. Participants were asked to continue their baseline concommitatnt medications related to COPD throughout the study.

### STUDY ENDPOINTS

The SGRQ-C questionnaire was administrered by phone by the CRA according to SGRQ-C guideline instructions with each subject.. SGRQ-C responses were recorded at baseline (day 0), day 30, and day 90 (final follow-up). The primary efficacy outcome(s) are changes from baseline to days 30 and 90 in the SGRQ-C total score.

### DATABASE MANAGEMENT

Clinical Research Associates validated all study data to insure data integrity and accuracy. All subjects’ de identified and validated data were then transferred via a secure transfer protocol process to the lead investigator for analysis and review. Data included demographics and SGRQ-C responses at each time point (baseline, 30, and 90 days). All data were converted to quantitative analysis at each timepoint using a Redcap-based scoring algorithm for SGRQ-C for both total and individual domain scores. The scoring algorithm provided by SGRQ-C was used to calculate questionnaire responses.

### STATISTICAL ANALYSIS

Descriptive statistics were computed for all study variables at each time point and aggregated across time points.

The primary efficacy endpoints and comparisons were changes from baseline to days 30 and 90 days for the quality-of-life questionnaire SGRQ-C (continuous measures). A paired t-test was used as primary efficacy analysis to assess changes from baseline for each of the outcomes. Dunnett’s multiple comparisons test was assessed to compare paired group means of SGRQ-C to baseline results at each timepoint.

To adjust for select independent predictors (age, gender, and others), multi-variable linear regression was used with day 30 or day 90 as the dependent variable and baseline and selected independent variables as predictors.

A linear mixed model (LMM) for the analysis of longitudinal data was used as a secondary analysis method to model continuous outcomes at baseline (day 0), day 30, and day 90 while simultaneously adjusting for gender, age, race, GOLD stage (i.e. E or B versus A), time, gender x time, and race x time. The models were used to assess longitudinal changes across time and allows adjusting for correlations between the repeated observations while adjusting for fixed and random effects. The fixed effects consisted of gender, age, race, GOLD stage, time, gender x time, and race x time. A random intercept was used to represent the subject-specific effect. The model building process was assessed in a 2-step fashion as outlined below.

1. Are there differences in slopes with respect to time?: To answer this question a “full model” with gender, age, race, GOLD stage, time, gender x time, race x time was used.
2. Then non-significant interaction terms were removed and a “reduced model” was used to assess main effects of gender, age, race, GOLD stage and time.

Finally, to assess the effect of gender and racial differences compared to baseline, a two-way ANOVA was analyzed with multiple comparisons test analyzed for statistical significance for gender differences (Tukey’s multiple comparisons test) and race (Dunnett’s Multiple Comparison test) at each time point compared to baseline.

### ETHICS APPROVALS

The study’s sponsor received a written opinion from Western Institutional Review Board’s (WIRB) IRB Affairs Department that the study’s design and protocol requirements, “meets the conditions for exemption under 45 CFR 46.101 (b)(4)”. WIRB’s conditions were that, “all of the data are in existence and the information will be recorded in such a manner that the subjects cannot be identified, directly or through identifiers linked to subjects and, that the results of the study will not be submitted to the FDA for marketing approval. Informed consent for participation was obtained from all patients. The protocol was also reviewed for reliance with the Insiitutional Review Board at the University of Alabama at Birmingham

### PERMISSIONS

A copyright license was obtained for the usage of the SGRQ-C questionnaire and its calculator scoring algorithm from the copyright owners at St. George’s University of London.

## Results

### DEMOGRAPHICS

We enrolled 102 subjects in the study. Baseline demographic data are summarized in Table 1. The Table indicates a representative distribution of age, gender, racial and ethnic variation amongst the overall cohort and indicates that appropriate sampling in this cohort was achieved to represent the usual COPD population. In addition, the patient population is mostly patients with advanced stage COPD (majority GOLD B or E) and many required oxygen therapy. As expected most patients were former or current smokers. Most patients (>88%) reported a singular pulmonary diagnosis of COPD only. The remainder reported physician diagnosis of asthma, former NTM infection, or “other dyspnea”. 100% of patients carried a diagnosis of COPD and no patients carried a physician assessed diagnosis of bronchiectasis based on CT scans reviewed by referring physicians before referral.

Patients baseline symptoms were recorded on the initial questionnaire and 77% of the patients reported cough most days of the week, 66% reported increased phlegm most days of the week, 74% reported having shortness of breath most days of the week, and 47% reported wheezing most days of the week. This indicates a relatively significant symptom burden amongst the study population prior to starting HFCWO therapy.

Concomittant medication at baseline are summarized in Supplemental Table 1. Most patients were not using mucolytic or airway clearance methods at baseline. However, the majority of patients were on guideline-based therapies for advanced stage COPD.

Compliance estimates indicate >90% compliance with the device.

### CHANGES IN QOL (OVERALL COHORT)

**Figure 1.**
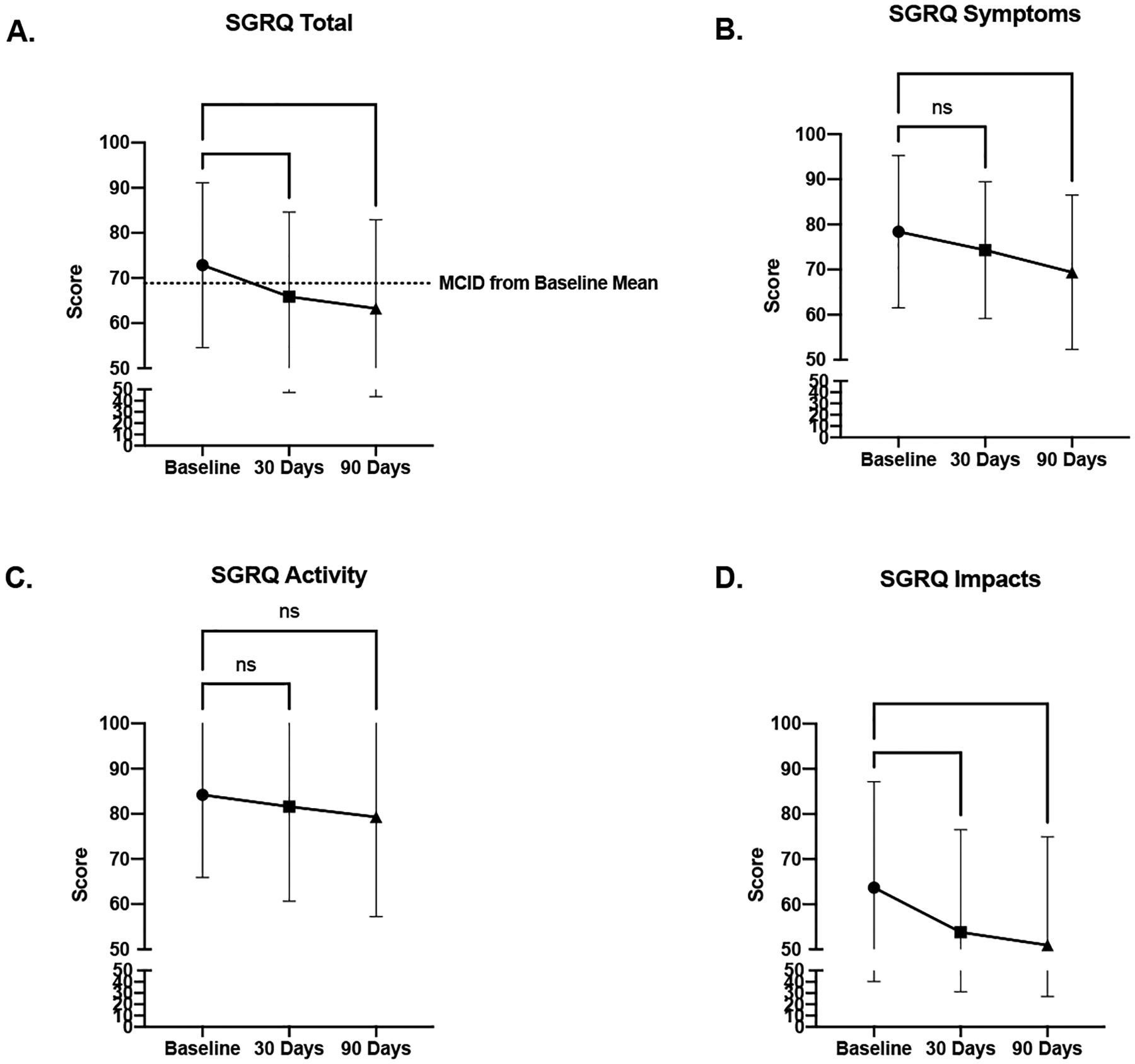
Change in SGRQ-C Score from Baseline in the overall cohort at 30- and 90-day timepoints. A. SGRQ “Total” Score change. Dashed line indicates MCID for this cohort from baseline (based on MCID of −4 points). Scoring of individual score components of the SGRQ-C was also analyzed for changes in B. SRGQ “Symptoms” score, C. Change in SGRQ “Activity” score, and D. Change in SGRQ “Impacts” Score. *p<0.05, **p<0.01, ***p<0.001 by one-way ANOVA with Dunnett’s multiple comparisons tests.

The primary endpoint of the study was the change in SGRQ-C score compared to baseline (72.84 [69.25,76.43 95% CI]). The overall cohort of patients demonstrated a significant reduction in the SGRQ-Cat 30 days (65.90 [62.13, 69.67 95% CI]) that was sustained at 90 days (63.26 [59.36,67.16 95% CI]) compared to baseline (Fig. 1 A). In addition, individual components of the SGRQ-C i.e., Symptoms, Activity and Impacts were analyzed (Table 3) and the changes were plotted indicating significant reductions at 90 days in the “Symptoms” and “Impacts” components compared to baseline (Fig. 1 B–D).

### GENDER OR RACE EFFECTS IN CHANGES IN QOL

Since the overall cohort demonstrated clinically and statistically significant improvements in QOL based on the SGRQ-C at 30 and 90 days, we sought to determine if race and/or gender were independent factors in the changes observed.

**Figure 2.**
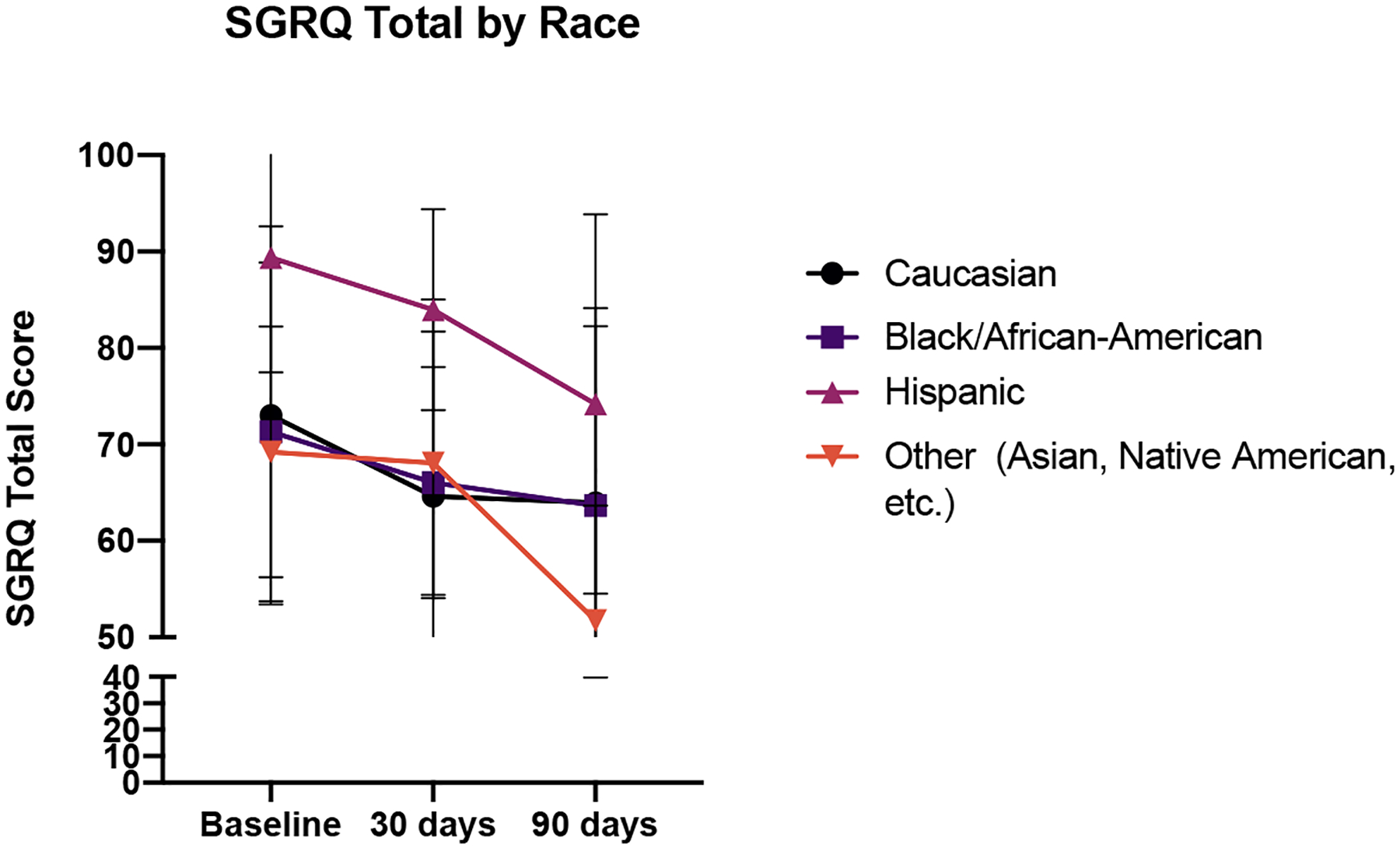
Assessment of SGRQ-C Total score at 30- and 90-day timepoints compared to baseline assorted on the basis of self-reported race and ethnicity. p=NS for all comparisons of racial and ethnic differences in change of score compared to baseline, despite the relatively higher baseline values for the self-reported “Hispanic” cohort ethnicity. All p-values assessed by 2-way ANOVA with Dunnett’s Multiple Comparisons test.

**Figure 3.**
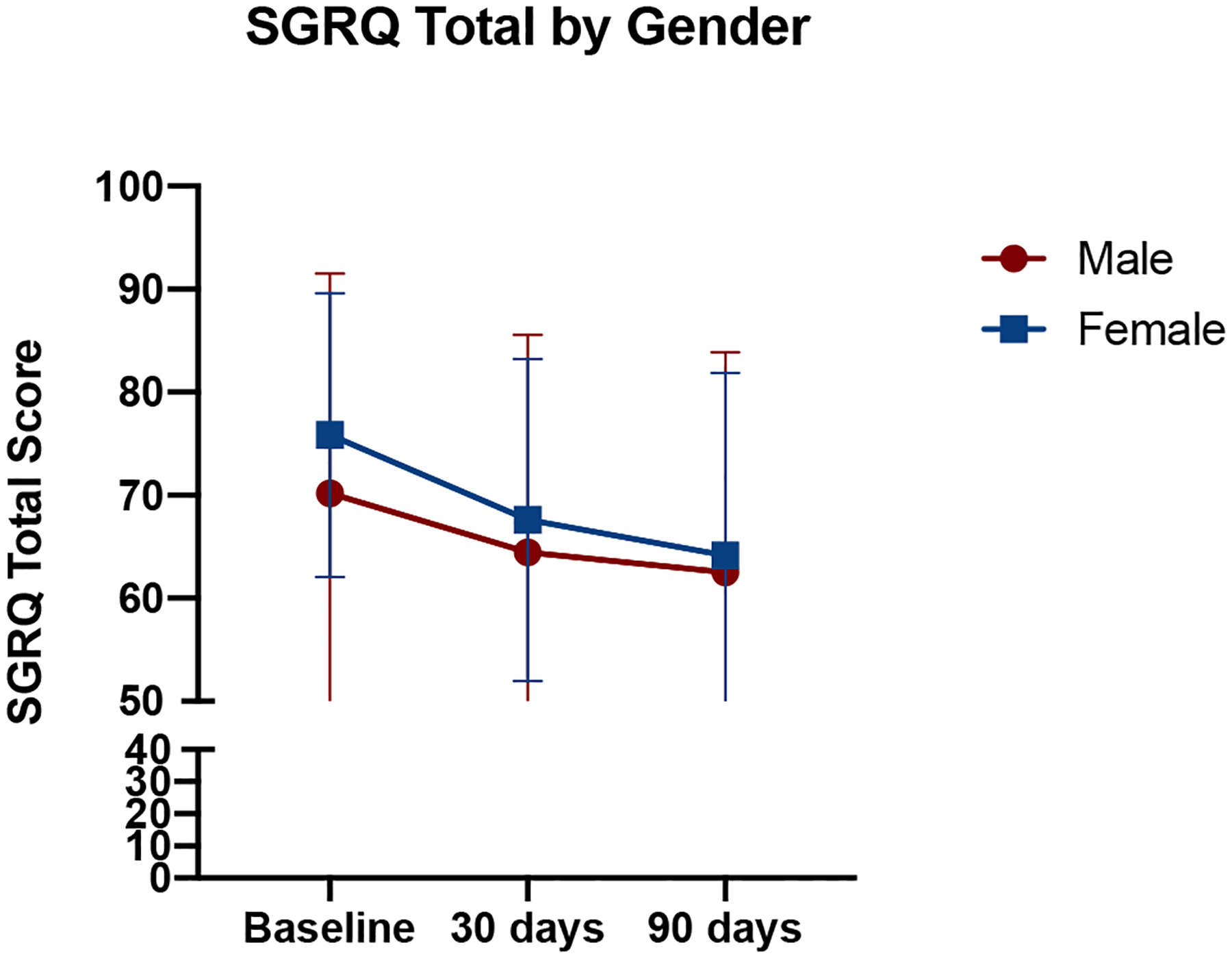
Assessment of SGRQ-C Total score at 30- and 90-day timepoints compared to baseline assorted based on self-reported gender classification. p=NS for all comparisons of gender difference in change of score compared to baseline. All p-values assessed by 2-way ANOVA with Tukey’s Multiple Comparisons test.

We first assorted the overall cohort based on self-reported race and ethnic classification and analyzed the differences in baseline and 30- and 90-day QOL timepoints as summarized in Figure 2. We then assorted the overall cohort based on self-reported gender and analyzed the same effects on the 30- and 90-day timepoints. Notably, no significant differences were seen in changes of QOL scores when comparing self-reported “female” vs “male” gender. No participants reported “non-binary” or “no gender”. This data is summarized in Figure 3.

To further confirm there is no interaction of gender or race/ethnicity and time, we analyzed the longitudinal outcomes to assess the effects of gender, age, race/ethnicity, time, gender x time, and race x time using a linear mixed model (LMM) in a two-step procedure. First, we asked if there were any differences in slopes in change of QOL data with respect to time. To answer that a “full model” multivariable model with gender, age, race, time, gender x time, race x time were analyzed. The interaction terms were not significant for any of the outcomes in the SGRQ-C “Total” or sub-scores (see Figures 2–3 and Table 2).

Finally, after removing interaction effects, we used a reduced multivariable model to assess the main effects of age, race/ethnicity, gender, and time on therapy on change in QOL assessments from baseline to 90 days. When the models were reduced, the only significant variable was “time on therapy” further indicating that the improvement of SGRQ-C Total, Activity, and Impact Scores, from baseline to 90 days is not based on gender, age, or racial effects in the cohort. For SGRQ-C Symptom Scores, both time and age were significant. These findings are summarized in Table 2 for the “Total” Score and for sub-scores in the data supplement (Supplementary tables 2–5).

Notably, GOLD stage (B or E versus A) did not have an effect In the overall LMM on total SGRQ-C score at any timepoint.

## Discussion

This open-label observational study demonstrates a significant improvement in QOL based on SGRQ-C scoring after using HFCWO as an adjunctive therapy for patients with a primary diagnosis of COPD without a physician diagnosis of concomitant bronchiectasis. Prior data have reported significant efficacy in improvements in lung function and inflammatory cells but have not explicity examined the effect of HFCWO on quality of life in COPD.^21^ In the same study, only a small cohort of patients had analysis of effects of HRQOL (CAT scores) when using HFCWO.^21^ A recent meta-analysis concluded that prior investigation of HFCWO was limited to conclude no significant effect on outcomes aside from the volume of sputum production and found no definitive efficacy in lung function or QOL.^22^ Subsequently, a randomized controlled trial investigated sixty patients with one arm on HFCWO and showed improvements in FEV_1_^23^.

Our study addresses these limitations in complete assessment of the effect of HFCWO on QOL. We demonstrate significant improvements in several component scores of the SGRQ-C including the “Impacts” and “Symptoms” score both of which were clinically significant (>MCID). This indicates that the improvement in quality of life is broader and may suggest benefits beyond clearance of pathological mucus alone, although this cannot be confirmed in this limited trial due to confounding. Thus this study adds to the literature the finding of initial improvement of QOL using HFCWO in patients broadly with COPD in a real-world pragmatic setting.

Furthermore, the study indicates that the initial effect of QOL was sustained at 90 days. This suggests meaningful longer-term response to HFCWO therapy. However, due to the limited study duration, we cannot conclude that HFCWO will have longer term effects. Furthermore, the open-label nature of the study creates some biases towards positive clinical responses, which cannot be controlled for in the analysis nor accounted for in the confounding. The controlled nature and patient characterization does indicate that the sustainment of symptom improvement is evidence that the treatment should be analzyed further.

Our study further demonstrates that the initial and longer-term improvements in QOL were observed in patients in all self-reported racial/ethnic groups and when divided based on self-reported gender. While we cannot definitively conclude that there are no racial, ethnic, or gender differences in response to HFCWO, this generates an intriguing hypothesis that this therapy may universally improve QOL in COPD patients. Finally multivariable regression modelling of the data suggests that time on therapy is the key variable for improvements in QOL, while also supporting the hypothesis that HFCWO therapy may work across a large spectrum of patients. Furthermore these responses were not unique to patients with more severe GOLD stage (B or E). This contrasts with other recent therapies with more limited indications such as macrolides, frequent exacerbators, or those with the chronic bronchitis phenotype^24,25^.

Mucus clearance is a critical and protective function of the airways and lungs. Failure of this function results in repeated lung infections and eventual respiratory insufficiency leading to increased morbidity and mortality^26^. COPD is the third leading cause of death in the United States and is increasing in incidence worldwide, therefore finding effective therapies targeted at improving mucus obstruction is vital to improving COPD morbidity and mortality^27^. Improving quality of life for the COPD patients has great implications. Unfortunately, COPD symptoms reduce the patient’s ability to perform activities of daily living potentially causing personal relationships and activities to become problematic^28^.

While this study was was intended to serve as a pragmatic study that recruited patients with a previous COPD diagnosis and were prescribed HFCWO airway clearance therapy, we again acknowledge several limitations in the study. First, the study was conducted as a small, open-label study. Therefore, we cannot definitively assess whether HFCWO initiation was the only variable resulting in improvements in QOL scores nor account for bias in a subjective patient repoted outcome measure. Furthermore, baseline exacerbation rate was not assessed so we cannot use this data to further assess efficacy. In addition, variations in the timing of other therapies was not accounted for in this study, but patients were asked to continue their baseline regimen of inhaled medications and other mucus clearance therapy. However, the symptoms burden was relatively high in many patients as evidence by the mean baseline “Total” and “Symptoms’ score. Therefore, we can reasonably conclude that many patients had productive cough and other symptoms that may be associated with mucus hypersecretion.

Future studies should randomize patients in a larger cohort to determine if HFCWO initiation is definitively associated with improved QOL. This study design would be aided by capturing baseline exacerbation rates to assess this key endpoint in trials aimed at improving mucus clearance.

## Figures and Tables

**Table 1.** Baseline Demographics of COPD Cohort Subjects (n=102 Subjects)

**Median Age, y.o. (Range)**	72 (37–92)
**Self-Reported Race (n, percentage of total)**	
*African-American*	9 (8.8)
*Caucasian (non-Hispanic)*	69 (67.6)
*Hispanic*	4 (3.9)
*Other (includes Asian and mixed race)*	20 (19.6)
**Gender (n, % of total)**	
*Female*	47 (46.1)
*Male*	55 (53.9)
**2024 GOLD Stage of Participants**	
*GOLD A*	10 (9.8)
*GOLD B*	48 (47.1)
*GOLD E*	44 (43.1)
**Oxygen Utulization**	
***Yes***	65 (63.7)
***No***	37 (36.3)
**Smoking Status**	
***Current***	13 (12.7)
***Former***	84 (82.4)
***Unknown***	5 (4.9)
**% with Secondary Pulmonary Diagnosis besides COPD( includes asthma,NTM, and other dyspnea**	12 (11.8%)

y.o. = years old, NTM = non-tuberculous mycobacterial infection

**Table 2.** SGRQ-C Total Score Final LMM Results

Effect	Estimate	Standard Error	DF	t Value	Pr > |t|
**Intercept**	95.8389	13.7954	101	6.95	<.0001
**Gender**	2.1524	3.5178	98.6	0.61	0.5421
**Age**	−0.3440	0.1832	102	−1.88	0.0633
**Race**	−0.4104	1.9891	251	−0.21	0.8367
**GOLD Stage**	−0.3319	2.1354	102	−0.32	0.5698
**Time**	−0.1037	0.01510	196	−6.87	<.0001

**Table 3.** SGRQ-C Total and Sub-Score

	Baseline (N=102)	30 days (N=97)	90 days (N=100)
**SGRQ Total**	72.84 [69.25, 76.43 95% CI]	65.90 [62.13, 69.67 95% CI]	63.26 [59.36, 67.16 95% CI]
**SGRQ Symptoms**	78.38 [75.05, 87.71 95% CI]	74.31 [71.27, 77.35 95% CI]	69.41 [66.05, 72.77 95% CI]
**SGRQ Impacts**	63.67 [59.04, 68.30 95% CI]	53.79 [49.33, 58.25 95% CI]	50.99 [46.29, 55.69 95% CI]
**SGRQ Activity**	84.19 [80.53, 87.85 95% CI]	81.60 [77.47, 85.73 95% CI]	79.29 [74.91, 83.67 95% CI]

## Data Availability

All data is housed in a locked database by the principal investigator. Data will be made available upon written request to the corresponsding author.

## Interpretation

This open-label observational study demonstrated initial and sustained improvements in quality of life when HFCWO was used as add on therapy for patients with a primary diagnosis of COPD without concomitant bronchiectasis. Further randomized trials are necsssary to assess the full effect of HFCWO on symptoms and outcomes in COPD.

## Take home points

Use of HFCWO vest therapy as add on therapy for patients with a primary diagnosis of COPD without concomitant bronchiectasis may improve QOL and is worthy of future studies to confirm efficacy on QOL.

## Abbreviations:

COPD: Chronic Obstructive Pulmonary Disease
CF: Cystic Fibrosis
HFCWO: High Frequency Chest Wall Oscillation
QOL: Quality of Life
SGRQ-C: The St. George’s Respiratory Questionnaire for COPD Patients
